# Supplementary material for: A conserved glycine harboring disease-associated mutations permits NMDA receptor slow deactivation and high Ca2+ permeability
Source: Nat Commun. 2018 Sep 14;9:3748. doi: 10.1038/s41467-018-06145-w (PMC6138751; doi:10.1038/s41467-018-06145-w)
Supplement: Supplementary file 1 — Supplementary Information [file 41467_2018_6145_MOESM1_ESM.pdf]

SUPPLEMENTARY MATERIAL

A conserved glycine harboring disease-associated mutations permits NMDA receptor slow deactivation and high Ca^{2+} permeability

Amin et al

Supplementary Table 1. *De novo* and inherited missense mutations in and around the GluN1, GluN2A, and GluN2B M4 transmembrane segments (relates to Figure 1). 2

Supplementary Figure 1. Sequence alignments of the M4 segments (relates to Figure 1). 3

Supplementary Table 2. Response of human wild-type GluN1/GluN2A (hN1/hN2A) and GluN1/GluN2B (hN1/hN2B) and NMDARs containing disease-associated mutations in the M4 segment to brief glutamate applications (relates to Figure 2). 4

Supplementary Figure 2. *de novo* missense mutations in the M4 segments alter NMDAR desensitization (relates to Figure 2). 5

Supplementary Figure 3. Additional examples of single channel recordings for human NMDARs containing disease-associated missense mutations (relates to Figure 3). 6

Supplementary Table 3. Single channel properties of human wild-type GluN1/GluN2A (hN1/hN2A) and GluN1/GluN2B (hN1/hN2B) and NMDARs containing disease-associated mutations in the M4 segment (relates to Figure 3 and Supplementary Figure 3). 7

Supplementary Table 4. Single channel properties of alanine substitutions at glycines in the M4 segments of rat GluN1 and GluN2A (rN1/rN2A) or human GluN1 and GluN2B (hN1/hN2B) (relates to Figure 5). 8

Supplementary Table 5. Closed state durations and occupancies for wild type and alanine substitutions of the conserved glycine in GluN1 and GluN2A (relates to Figure 6).................. 9

Supplementary Table 6. Open state durations and occupancies for wild type and alanine substitutions of the conserved glycine in GluN1 and GluN2A (relates to Figure 6)................10

Supplementary Figure 4. Glycine-to-alanine at the conserved glycine primarily disrupt pore structure (relates to Figures 6 & 7). 11

Supplementary Figure 5. Comparison of glutamate and glycine deactivation rates in wild type and alanine substitutions of the conserved glycine (relates to Figure 6). 12

Supplementary Figure 6. Global changes in RMSD (relates to Figure 7). 13

Supplementary Table 1. ***De novo*** **and inherited missense mutations in and around the GluN1, GluN2A, and GluN2B M4 transmembrane segments (relates to Figure 1).**

GluN1

Protein Mutation	# of patients	Age at seizure onset	Clinical Phenotype*	References
Gly815Arg	3	2-12 mo	Epilepsy, Severe ID/DD, Dyskinesia, Hypotonia, Cerebral atrophy	Ohba et al., 2015[1]<br>Lemke et al., 2016[2]
Gly815Val	1	7 mo	Epilepsy, Severe ID/DD, ASD, Dyskinesia, Hypotonia, Cerebral atrophy	Lemke et al., 2016[2]
Phe817Leu	2	no seizures	Severe ID/DD, ASD, Hypotonia	Lemke et al., 2016[2]
Gly827Arg	3	no seizure – 5 yr	Epilepsy, Severe ID/DD, ASD, Dyskinesia, Hypotonia	Lemke et al., 2016[2]

GluN2A

Protein Mutation	# of patients	Age at seizure onset	Clinical Phenotype	References
Leu812Met	1	6.5 yr	Epilepsy, ID/DD	Pierson et al., 2014[3]
Ile814Thr	1	5 yr	Epilepsy	Lemke et al., 2013[4]
Met817Val	1	14 mo	Epilepsy, ID/DD, ASD	Venkateswaran et al., 2014[5]

GluN2B

Protein Mutation	# of patients	Age at seizure onset	Clinical Phenotype	References
Met818Leu	1	unknown	ID/DD	Platzer et al., 2017[6]
Met818Thr	1	2 mo	Epilepsy, DD, Hypotonia	Platzer et al., 2017[6]
Ala819Thr	1	unknown	ID/DD	Platzer et al., 2017[6]
Gly820Ala	5	no seizure – 1 yr	Epilepsy, ID/DD, ASD, Hypotonia, Cerebral Atrophy	Platzer et al., 2017[6]
Gly820Val	1	unknown	ID/DD	Platzer et al., 2017[6]
Gly820Glu	1	unknown	Severe ID/DD, Cerebral Atrophy	Hamdan et al., 2014[7]
Met824Arg	1	no seizures	Severe ID/DD, Dyskinesia	Zhu et al., 2015[8]
Leu825Val	1	no seizures	ASD	Tarabeux et al., 2011[9]
Gly826Glu	1	no seizures	Moderate ID/DD	Platzer et al., 2017[6]

*ID= intellectual disability, DD = developmental delay, ASD = Autism spectrum disorder

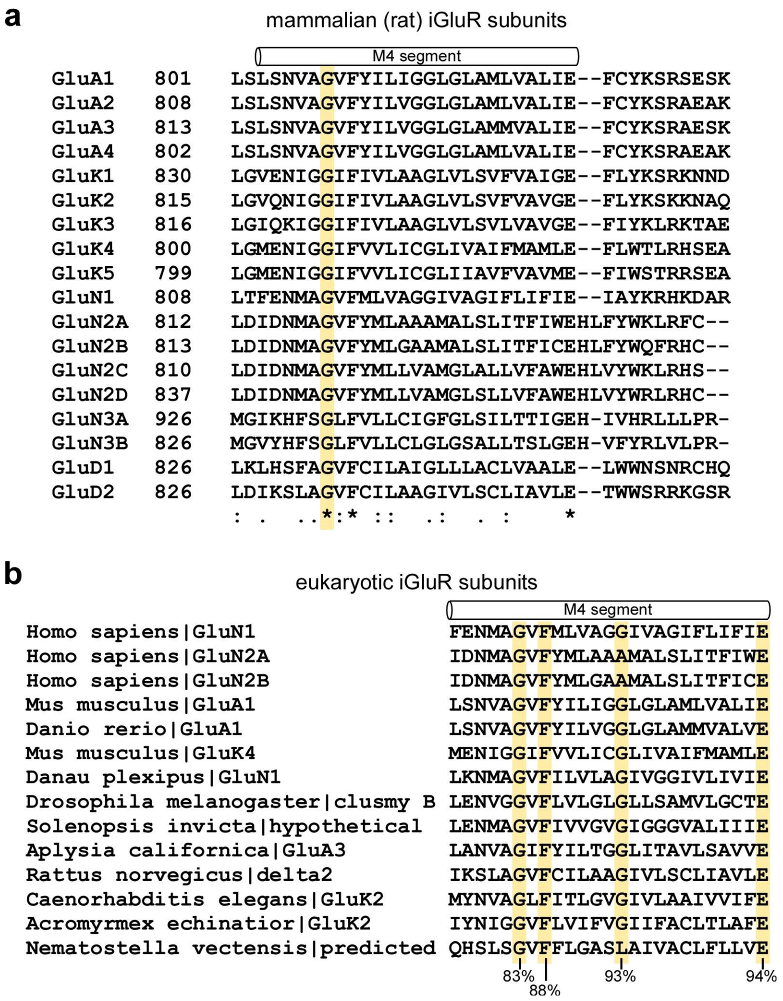

Supplementary Figure 1. Sequence alignments of the M4 segments (relates to Figure 1).

(a) Alignment of the M4 segments and residues on the N- and C-terminal sides for rat ionotropic glutamate receptor subunits using Clustall. Only three residues, a glycine (G), a phenylalanine (F), and a glutamate (E), are completely conserved across all subunits (*asterisks*). Missense mutations are present at the conserved glycine in GluN1 (G815R, G815V) and GluN2B (G820A, G820V, G820E) subunits as well as the phenylalanine in GluN1 (F817L). Positions showing various degree of conservation, in terms of the nature of the side chain, are indicated with a colon (high similarity) or a period (less similarity).

(**b**) Alignment of the M4 segments in an extensive sequence alignment of eukaryotic iGluRs [10]. Of the 4604 positions in the sequence alignment, only 32 showed a consensus score greater than 80%, four of which are present in the M4 segment: glycine (83%), phenylalanine (88%) and glutamate (94%) as in rat iGluRs (Supplementary Figure 1A). In addition, another position is also highly conserved, 93%, with the side chains at this position typically small (glycine or alanine) presumably because it is involved in transmembrane interactions[11]. For details on approach to generate the multiple sequence alignment see Alsaloum et al., 2016.

Supplementary Table 2. Response of human wild-type GluN1/GluN2A (hN1/hN2A) and GluN1/GluN2B (hN1/hN2B) and NMDARs containing disease-associated mutations in the M4 segment to brief glutamate applications (relates to Figure 2).

Construct	I_{peak} *pA*	rise time *ms*	τ_{fast} *ms*	A_{fast} *%*	τ_{slow} *ms*	A_{slow} *%*	$\tau_{weighted}$ *ms*	n
**hN1/hN2A**	-780 ± 70	5.7 ± 0.1	39 ± 1	69 ± 2	180 ± 5	31 ± 2	81 ± 2	11
hN1(G815V)	-80 ± 15*	2.8 ± 0.3*	20 ± 2*	64 ± 5	74 ± 10*	36 ± 5	35 ± 4*	5
hN1(G815R)	-70± 20*	3.0 ± 0.3*	20 ± 1*	59 ± 4	84 ± 8*	41 ± 4	39 ± 2*	6
hN1(F817L)	-285 ± 30	4.8 ± 0.1*	24 ± 2*	78 ± 2	110 ± 7*	22 ± 2	42 ± 3*	6
hN1(G827R)	*nd*							
hN2A(I814T)	-460 ± 110	7.1 ± 0.4	49 ± 4	74 ± 5	230 ± 20	26 ± 5	97 ± 13	5
hN2A(M817V)	-250 ± 50	8.3 ± 0.6	370 ± 40^	44 ± 5	3360 ± 460^	56 ± 5	1970 ± 320^	5
**hN1/hN2B**	-130 ± 10	10.6 ± 0.5	260 ± 20	52 ± 1	1090 ± 40	48 ± 1	660 ± 30	9
hN1(G815R)	-40 ± 4*	3.5 ± 0.2*	46 ± 4*	43 ± 2	430 ± 30*	57 ± 2	250 ± 10*	5
hN2B(G820A)	-51 ± 10	5.4 ± 0.5*	89 ± 9*	66 ± 2	350 ± 30*	34 ± 2	205 ± 10*	7
hN2B(G820E)	*nd*							
hN2B(L825V)	-58 ± 6	7.2 ± 0.5	210 ± 30	40 ± 3	1260 ± 160	60 ± 3	800 ± 90	7
hN2B(G826E)	-63 ± 8	14.8 ± 1.0	250 ± 20	47 ± 5	2560 ± 100*	53 ± 5	1390 ± 80^	6

Values shown are mean ± SEM. Whole-cell currents were generated in response to 2 ms glutamate applications. The rise time is the 10-90% of the rising phase of current. The decay component was fit with a double exponential with τ_{fast} and τ_{slow} and A_{fast} and A_{slow} the τs and %areas of the fast and slow components, respectively.
nd, no glutamate-activated current was detected in the whole-cell mode either using brief (2 ms) or sustained (2.5 s) glutamate applications.
Tagged values are significantly less (*) or greater (^) than wild type (*$p < 0.05$, two-tailed Student's t-test, unpaired*).

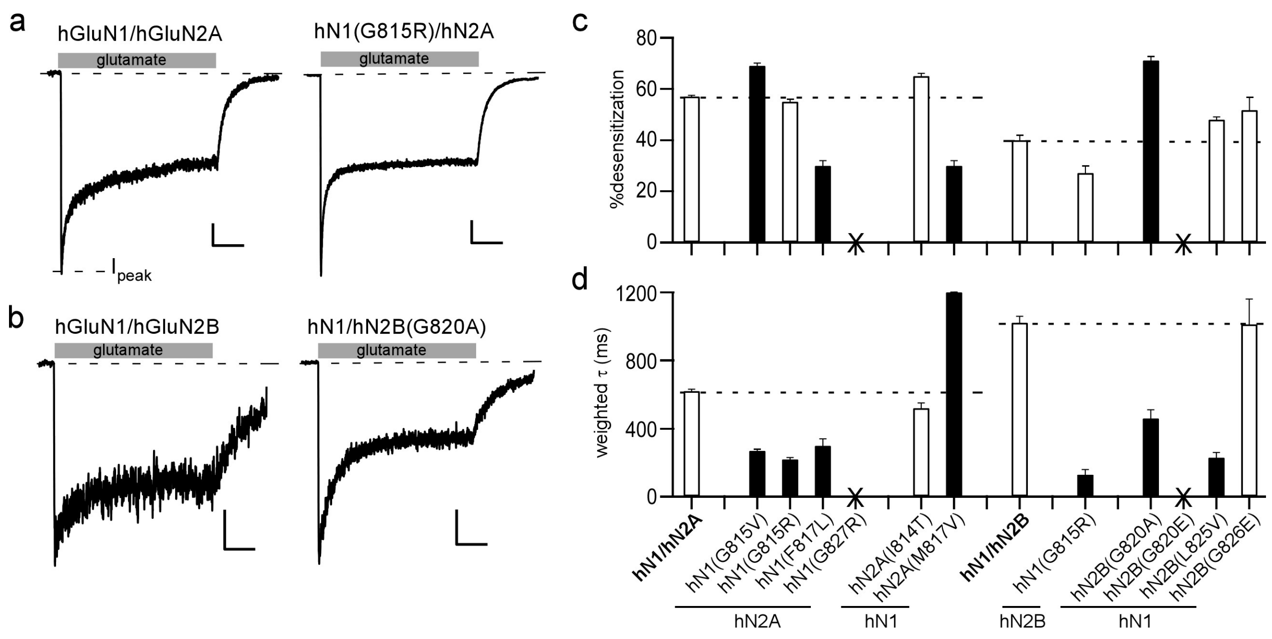

Supplementary Figure 2. ***de novo*** **missense mutations in the M4 segments alter NMDAR desensitization (relates to Figure 2).**

(a & b) Whole-cell glutamate-activated currents recorded in response to sustained (2.5 sec) glutamate application (1 mM, gray bar) to measure NMDAR desensitization. Current amplitudes were measured either at the peak (I_{peak}) or near the end of the glutamate application when currents reached steady-state (I_{ss}). Holding potential, -70 mV. Scale bars: (a) 100 pA, wild type; 50 pA, hN1(G815R); (b) 50 pAs for both; time base was 0.5 sec for all.

(**c & d)** Bar graphs (mean ± SEM) (n $\geq$ 4) showing %desensitization (**c**) or rate of desensitization (weighted tau) (**d**). Solid bars indicate values significantly different from their respective wild types (*p < 0.05, t-test*). For hN1(G827R)/hN2A or hN1/hN2B(G820E), no glutamate-activated current was detected in the whole-cell mode either using brief (2 ms) (Figure 2, Supplementary Table 2) or sustained (2.5 s) glutamate applications.

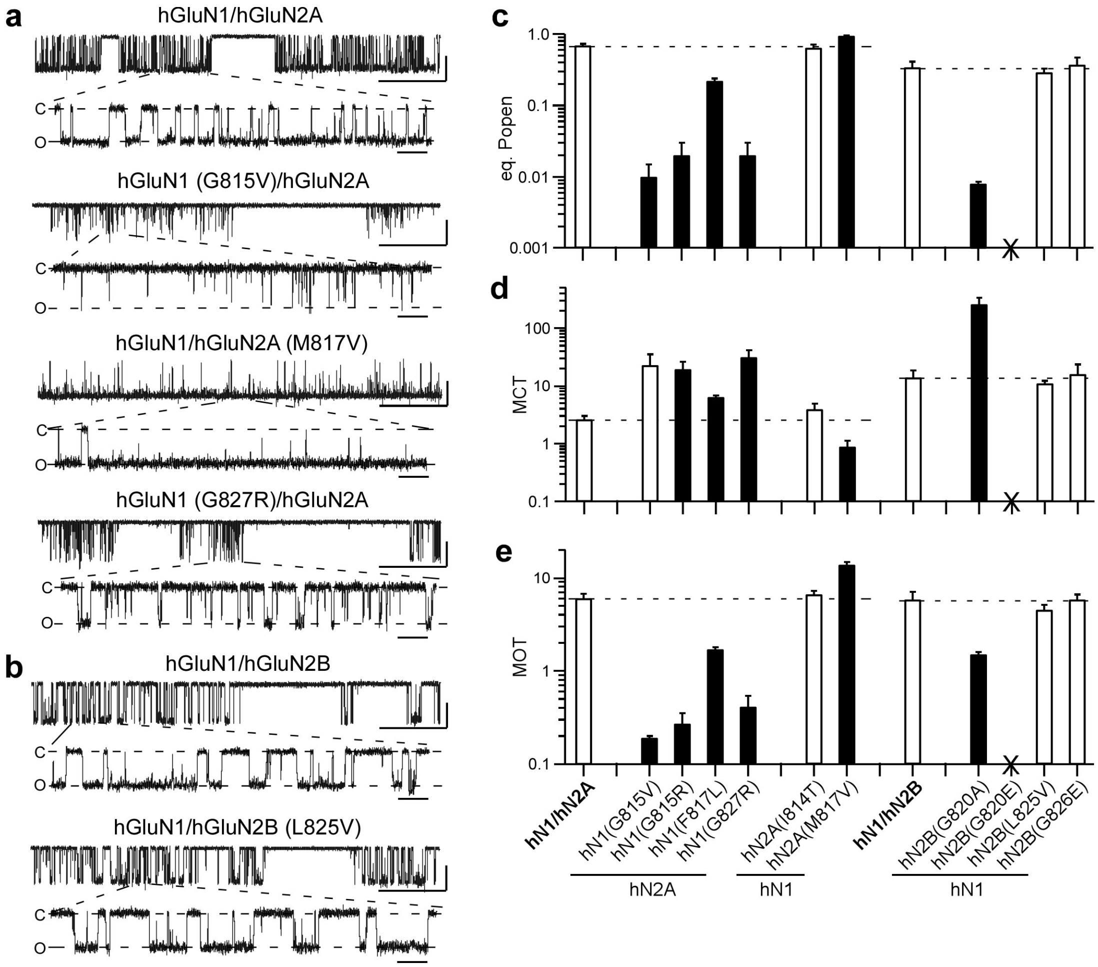

Supplementary Figure 3. Additional examples of single channel recordings for human NMDARs containing disease-associated missense mutations (relates to Figure 3).

(a & b) Example single channel recordings of wild type human GluN1/GluN2A (hGluN1/hGluN2A) (**a**) and GluN1/GluN2B (hGluN1/hGluN2B) (**b**) and the same constructs containing disease-associated missense mutations in the M4 transmembrane segments. Recordings were generated and displayed as in Figure 3a. Scale bars: 5 pAs for all; time base is 500 ms (upper trace for each construct) and 20 ms (lower trace for each construct).

(c-e) Bar graphs (mean ± SEM) showing equilibrium open probability (*eq.* P_o) **(c)**, mean closed time (MCT) **(d)**, and mean open time (MOT) (**e**) for wild type and various missense mutations in M4 (Supplementary Table 3). Solid bars indicate values significantly different from wild type ($p < 0.05$, *t-test*). No single channel (or whole-cell current, Figure 2, Supplementary Table 2) activity could be detected for hN1/hN2B(G820E).

Supplementary Table 3. Single channel properties of human wild-type GluN1/GluN2A (hN1/hN2A) and GluN1/GluN2B (hN1/hN2B) and NMDARs containing disease-associated mutations in the M4 segment (relates to Figure 3 and Supplementary Figure 3).

Construct	Total events (# of patches)	*i* *pA*	eq. P_{open}	MCT *ms*	MOT *ms*
**hN1/hN2A**	259972 (6)	-6.2 ± 0.6	0.69 ± 0.04	2.6 ± 0.4	6.0 ± 0.8
hN1(G815V)/hN2A	210540 (4)	-5.5 ± 0.2	0.01 ± 0.005*	22.8 ± 12.5	0.19 ± 0.01*
hN1(G815R)/hN2A	281957 (5)	-3.7 ± 0.5*	0.02 ± 0.01*	19.4 ± 6.7^	0.27 ± 0.08*
hN1(F817L)/hN2A	722027 (4)	-9.3 ± 0.5^	0.22 ± 0.02*	6.3 ± 0.5*	1.7 ± 0.1*
hN1(G827R)/hN2A	76342 (4)	-6.8 ± 0.6	0.02 ± 0.01*	31.3 ± 10.1^	0.41 ± 0.13*
hN1/hN2A(I814T)	444446 (5)	-7.5 ± 0.5	0.64 ± 0.07	3.9 ± 1.0	6.6 ± 0.6
hN1/hN2A(M817V)	372998 (5)	-6.5 ± 0.2	0.94 ± 0.01^	0.88 ± 2.4*	13.9 ± 1.1^
**hN1/hN2B**	209148 (5)	-7.1 ± 0.7	0.34 ± 0.07	14.0 ± 4.7	5.8 ± 1.2
hN1/hN2B(G820A)	29991 (4)	-9.2 ± 0.6	0.008 ± 0.0005*	256.2 ± 78.7^	1.5 ± 1.1*
hN1/hN2B(G820E)	*nd*				
hN1/hN2B(L825V)	144963 (4)	-7.2 ± 0.3	0.29 ± 0.04	11.1 ± 1.3	4.5 ± 0.6
hN1/hN2B(G826E)	54811 (5)	-8.4 ± 0.3	0.37 ± 0.10	16.0 ± 7.7	5.8 ± 0.8

Values shown are mean ± SEM for single-channel current amplitude (*i*), equilibrium open probability (P_o), mean closed time (MCT), and mean open time (MOT). Single channel currents were recorded in the on-cell mode at approximately -100 mV and analyzed in QuB (see Online Methods). Number of patches is in parenthesis to the right of total events. Eq P_o is the fractional occupancy of the open states in the entire single-channel recording, including long lived closed states. All data were idealized and fit at a dead time of 20 µs.
Tagged values are significantly less (*) or greater (^) than wild type (*$p < 0.05$, two-tailed Student's t-test, unpaired*).
nd, no glutamate-activated currents were detected in the whole-cell or on-cell mode.

Supplementary Table 4. Single channel properties of alanine substitutions at glycines in the M4 segments of rat GluN1 and GluN2A (rN1/rN2A) or human GluN1 and GluN2B (hN1/hN2B) (relates to Figure 5).

**Construct**	**Total events (# of patches)**	***i*** ***pA***	**eq. P_{open}**	**MCT** ***ms***	**MOT** ***ms***
rN1/rN2A	2108456 (14)	-7.1 ± 0.1	0.69 ± 0.03	2.5 ± 0.2	6.0 ± 0.5
rN1(G815A)/rN2A	121190 (6)	-6.1 ± 0.4	0.01 ± 0.005*	37.7 ± 7.3^	0.30 ± 0.01*
rN1(G822A)/rN2A	345225 (4)	-7.4 ± 0.3	0.81 ± 0.01^	2.08 ± 0.08	9.1 ± 0.8*
rN1(G823A)/rN2A	191681 (4)	-6.6 ± 0.3	0.51 ± 0.05*	5.5 ± 1.2	5.6 ± 0.8
rN1(G827A)/rN2A	366103 (4)	-8.1 ± 0.4	0.68 ± 0.09	2.4 ± 0.6	5.5 ± 1.1
rN1/rN2A(G819A)	65033 (4)	-6.7 ± 0.4	0.02 ± 0.01*	120.2 ± 27.9^	2.4 ± 0.2*
hN1/hN2B	209148 (5)	-7.1 ± 0.7	0.34 ± 0.07	14.0 ± 4.7	5.8 ± 1.2
hN1/hN2B(G820A)	29991 (4)	-9.2 ± 0.6	0.008 ± 0.0005*	256.2 ± 78.7^	1.5 ± 1.1*
hN1/hN2B(G826A)	97267 (4)	-7.6 ± 0.2	0.23 ± 0.10	18.5 ± 4.3	4.7 ± 0.5

Values shown and analyzed as in Supplementary Table 3.
Tagged values are significantly less (*) or greater (^) than wild type (*$p < 0.05$, two-tailed Student's t-test, unpaired*).

Supplementary Table 5. Closed state durations and occupancies for wild type and alanine substitutions of the conserved glycine in GluN1 and GluN2A (relates to Figure 6).

**Construct**		C_1	C_2	C_3	C_4	C_5
**N1/N2A**	τ (ms)	0.16 ± 0.01	1.3 ± 0.1	3.4 ± 0.2	17.7 ± 2.2	990 ± 90
	α (%)	46 ± 2	33 ± 2	20 ± 1	2.1 ± 0.1	0.1 ± 0.05
	n	10	10	10	10	10
**N1(G815A)**	τ (ms)	0.30 ± 0.04*	9.4 ± 4.2	32 ± 10*	210 ± 120	1070 ± 300
	α (%)	12 ± 8*	30 ± 10	57 ± 8*	9 ± 3	0.6 ± 0.2
	n	5	5	5	5	5
**N2A(G819A)**	τ (ms)	0.20 ± 0.01	6.5 ± 2.4	24 ± 6*	450 ± 360	4000 ± 1050
	α (%)	19 ± 1*	19 ± 6	50 ± 5*	9 ± 4	2.4 ± 0.1*
	n	4	4	4	4	4

Mean values (± SEM) for closed state durations (τ, ms) and occupancies (α, %). Values were derived after fitting idealized single-channel records to a 4- to 5-closed and 2- to 4-open state kinetic scheme (see Online Methods). Optimal fits were defined by log-liklihoods (see Online Methods). For closed and open time histograms, we analyzed records only if they contained > 10,000 events.
Asterisks indicate significance relative to GluN1/GluN2A ($p < 0.05$, *t-test*).

Supplementary Table 6. Open state durations and occupancies for wild type and alanine substitutions of the conserved glycine in GluN1 and GluN2A (relates to Figure 6).

**Construct**		O_1	O_2	O_3	O_4
**N1/N2A**	τ (ms)	0.15 ± 0.01	4.1 ± 0.4	8.7 ± 0.6	18.0 ± 3.2
	α (%)	16 ± 1	37 ± 10	39 ± 10	11 ± 5
	n	10	10	10	7
**N1(G815A)**	τ (ms)	0.17 ± 0.01	0.51 ± 0.03*	3.5 ± 1.6	
	α (%)	57 ± 3*	42 ± 3	1 ± 0.5	
	n	5	5	2	
**N2A(G819A)**	τ (ms)	0.13 ± 0.01	2.5 ± 0.4*	4.9 ± 1.0*	
	α (%)	27 ± 4	40 ± 9	31 ± 10	
	n	4	4	3	

Mean values (± SEM) for open state durations (τ, ms) and occupancies (α, %). See legend to Supplementary Table 5 for details.
Asterisks indicate significance relative to GluN1/GluN2A (*p < 0.05, t-test*).

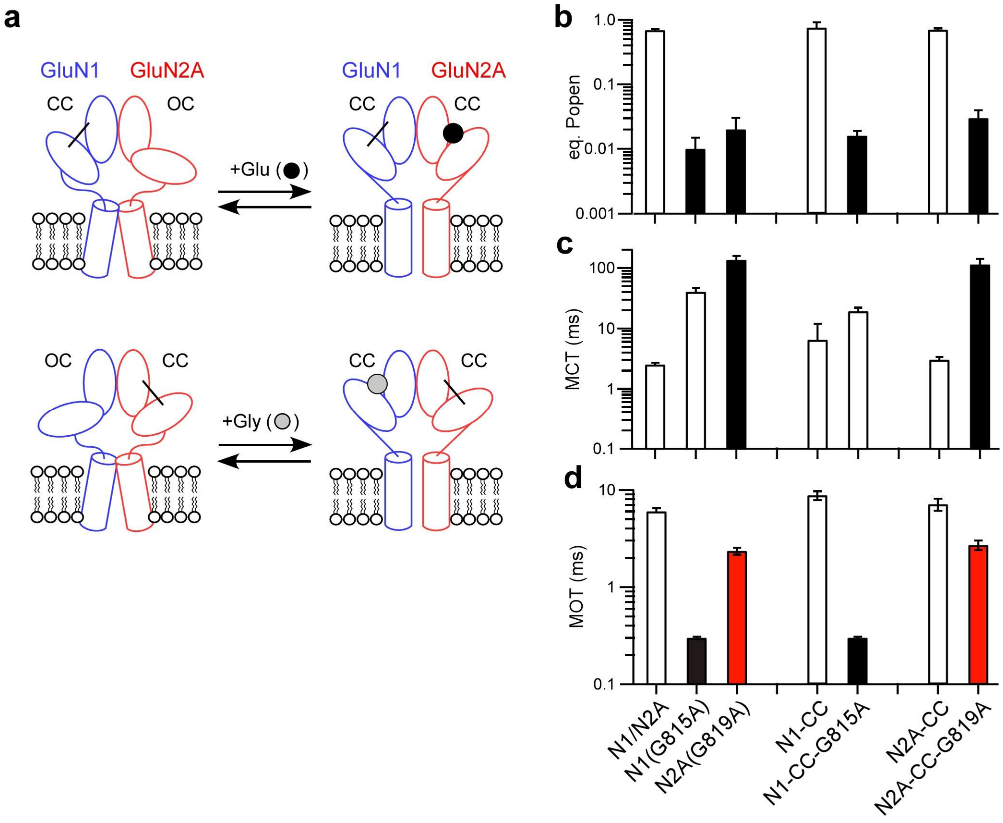

Supplementary Figure 4. Glycine-to-alanine at the conserved glycine primarily disrupt pore structure (relates to Figures 6 & 7).

Forcing the LBD into a closed clamshell configuration does not recover gating in the glycine-to-alanine background in either GluN1 or GluN2A, indicating that constraining this glycine does not greatly affect LBD stability (clamshell closure).

(**a**) NMDAR LBDs were closed using two engineered cysteines (CC) across the cleft in either GluN1 or GluN2A[12,13]. With these cysteines or in the presence of the appropriate agonist, the clamshell cleft is assumed to be closed (CC), whereas in their absence or in the absence of the appropriate agonist, the clamshell cleft is open (CO). Single-channel activity was recorded in the cell-attached configuration. For GluN1(CC)/GluN2A (*upper panel*), the pipette solution contained glutamate (1 mM) with no added glycine. For GluN1/GluN2A(CC) (*lower panel*), the pipette solution contained glycine (0.1 mM) with no added glutamate.

(**b-d**) Bar graphs (mean ± SEM)($n \geq 3$) showing equilibrium open probability (*eq.* P_o) **(b)**, mean closed time (MCT) **(c)**, and mean open time (MOT) (**d**) for wild type, GluN1(G815A)/GluN2A, and GluN1/GluN2A(G819A) (left three bars; Figure 5 and Supplementary Table 4) as well as G815A in the GluN1-CC background (center two bars) and G819A in the GluN2A-CC background (right two bars). Values for like manipulations in different backgrounds were not statistically different (*$p <$ 0.05, ANOVA, Tukey*).

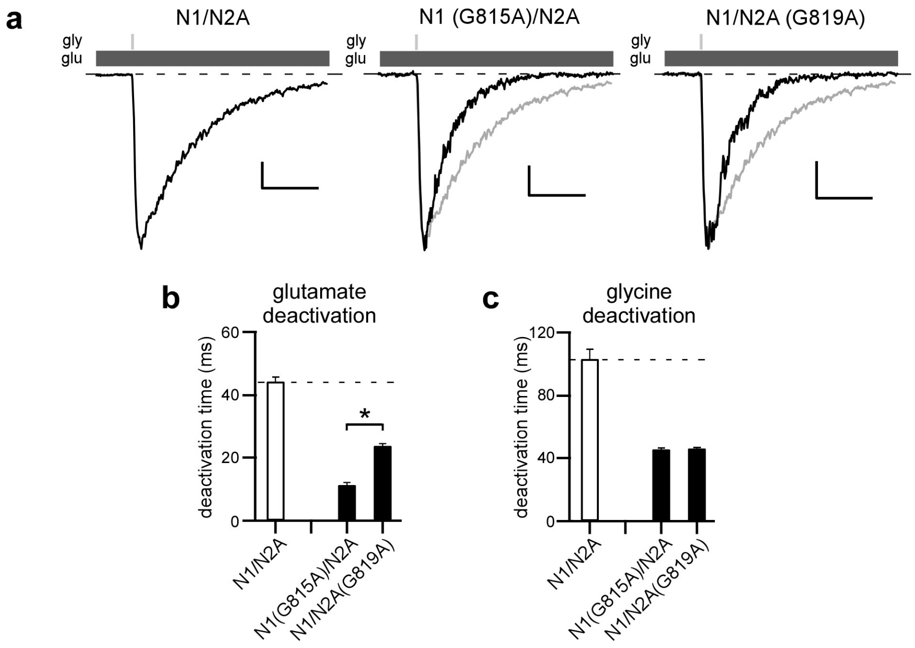

Supplementary Figure 5. Comparison of glutamate and glycine deactivation rates in wild type and alanine substitutions of the conserved glycine (relates to Figure 6).

(a) Whole-cell glutamate-activated currents from HEK 293 cells expressing wild-type (*left*) or alanine substitutions at the conserved glycine in GluN1 [N1(G815A)/N2A] (*middle*) or GluN2A [N1/N2A(G819A)] (*right*). Currents were recorded and analyzed as in Figures 2a & 2b except that glycine was applied briefly in the continuous presence of glutamate. Scale bars: 100 pA, wild type; 15 pA, N1(G815A); 50 pA, N2A(G819A); time base is 100 ms for all.

(**b & c**) Bar graphs (mean ± SEM)($n \geq 3$) showing deactivation rates either for glutamate (**b**)(same as in Figure 6e) or glycine **(c)**. Black bars indicate values significantly different from wild type whereas asterisks indicate values significantly different from each other (*$p < 0.05$, ANOVA, Tukey*).

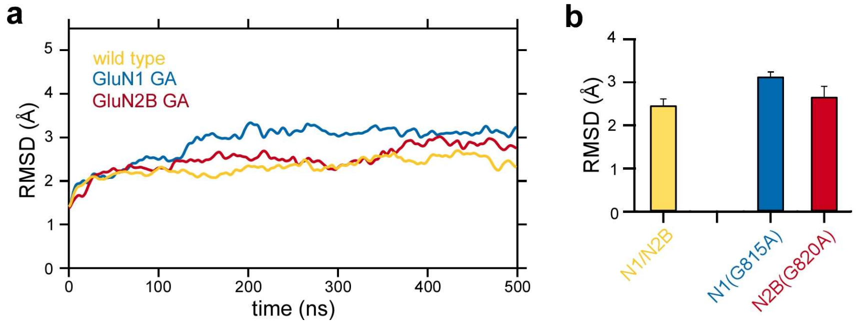

Supplementary Figure 6. Global changes in RMSD (relates to Figure 7).

(a) Cα RMSD of the TMD (M1-M4) helices as a function of MD simulation time for open state models: wild type GluN1/GluN2B, light orange; GluN1(G815A)/GluN2B, light blue; GluN1/GluN2B(G820A), maroon.

(b) Bar graphs showing average RMSD over 250-500 ns.

Supplementary References

1 Ohba, C. *et al.* GRIN1 mutations cause encephalopathy with infantile-onset epilepsy, and hyperkinetic and stereotyped movement disorders. *Epilepsia* **56**, 841-848, doi:10.1111/epi.12987 (2015).

2 Lemke, J. R. *et al.* Delineating the GRIN1 phenotypic spectrum: A distinct genetic NMDA receptor encephalopathy. *Neurology* **86**, 2171-2178, doi:10.1212/WNL.0000000000002740 (2016).

3 Pierson, T. M. *et al.* GRIN2A mutation and early-onset epileptic encephalopathy: personalized therapy with memantine. *Annals of clinical and translational neurology* **1**, 190-198, doi:10.1002/acn3.39 (2014).

4 Lemke, J. R. *et al.* Mutations in GRIN2A cause idiopathic focal epilepsy with rolandic spikes. *Nature genetics* **45**, 1067-1072, doi:10.1038/ng.2728 (2013).

5 Venkateswaran, S. *et al.* Whole-exome sequencing in an individual with severe global developmental delay and intractable epilepsy identifies a novel, de novo GRIN2A mutation. *Epilepsia* **55**, e75-79, doi:10.1111/epi.12663 (2014).

6 Platzer, K. *et al.* GRIN2B encephalopathy: novel findings on phenotype, variant clustering, functional consequences and treatment aspects. *J Med Genet* **54**, 460-470, doi:10.1136/jmedgenet-2016-104509 (2017).

7 Hamdan, F. F. *et al.* De novo mutations in moderate or severe intellectual disability. *PLoS Genet* **10**, e1004772, doi:10.1371/journal.pgen.1004772 (2014).

8 Zhu, X. *et al.* Whole-exome sequencing in undiagnosed genetic diseases: interpreting 119 trios. *Genet Med* **17**, 774-781, doi:10.1038/gim.2014.191 (2015).

9 Tarabeux, J. *et al.* Rare mutations in N-methyl-D-aspartate glutamate receptors in autism spectrum disorders and schizophrenia. *Transl Psychiatry* **1**, e55, doi:10.1038/tp.2011.52 (2011).

10 Alsaloum, M., Kazi, R., Gan, Q., Amin, J. & Wollmuth, L. P. A Molecular Determinant of Subtype-Specific Desensitization in Ionotropic Glutamate Receptors. *J Neurosci* **36**, 2617-2622, doi:10.1523/JNEUROSCI.2667-15.2016 (2016).

11 Salussolia, C. L. *et al.* Interaction of the M4 segment with other transmembrane segments is required for surface expression of mammalian alpha-amino-3-hydroxy-5-methyl-4-isoxazolepropionic acid (AMPA) receptors. *J Biol Chem* **286**, 40205-40218, doi:10.1074/jbc.M111.268839 (2011).

12 Blanke, M. L. & VanDongen, A. M. Constitutive activation of the N-methyl-D-aspartate receptor via cleft-spanning disulfide bonds. *J Biol Chem* **283**, 21519-21529, doi:M709190200 [pii]10.1074/jbc.M709190200 (2008).

13 Kussius, C. L. & Popescu, G. K. NMDA receptors with locked glutamate-binding clefts open with high efficacy. *J Neurosci* **30**, 12474-12479, doi:10.1523/JNEUROSCI.3337-10.2010 (2010).
